# Access to and quality of contraceptive healthcare for undocumented immigrant women in California

**DOI:** 10.1016/j.ssmqr.2025.100655

**Published:** 2025-10-24

**Authors:** Rebecca Woofter, May Sudhinaraset

**Affiliations:** a University of California Los Angeles Fielding School of Public Health, Department of Community Health Sciences, US; b Rutgers School of Public Health, Department of Health Behavior, Society, and Policy, US

**Keywords:** Contraception, Contraceptive healthcare, Undocumented immigrants, Healthcare barriers, Healthcare quality

## Abstract

Immigrants have lower rates of contraceptive use compared to non-immigrants. Undocumented immigrants face multiple barriers to healthcare and often experience poor-quality healthcare, yet few studies have examined contraceptive healthcare in this population. This study aimed to explore access to and quality of contraceptive healthcare among undocumented immigrants. Twenty-four in-depth interviews were conducted between April and July 2022 with Asian and Latinx undocumented immigrant women of reproductive age living in California within the United States. Interviews were inductively coded and themes were identified through thematic analysis. Most participants were aged 25–29, had college degrees, and were Deferred Action for Childhood Arrivals (DACA) recipients. All participants had ever used some form of contraception including condoms. Access to contraceptive healthcare was primarily impacted by knowledge about contraception, health insurance coverage, and knowledge about using health insurance. Participants who accessed contraceptive healthcare described a range of experiences, including those that were judgmental or dismissive, cursory or impersonal, or person-centered. This study highlights barriers to contraceptive healthcare and aspects of poor-quality contraceptive healthcare among undocumented immigrants, even in a highly-educated sample of mostly DACA recipients living in an inclusive state. Undocumented immigrants with lower socioeconomic status, without the protection of DACA, and living in more exclusive state policy climates may face additional barriers to contraceptive healthcare and worse quality of care. Efforts are needed to improve contraceptive education, consistent health insurance coverage and knowledge, and to ensure person-centered contraceptive healthcare for undocumented immigrants. These efforts will help work towards reproductive justice in this population.

## 1. Introduction

In the United States (U.S.), nearly half of pregnancies are unintended, either not wanted at all or wanted at a later time (Finer & Zolna, 2016). Unintended pregnancies are associated with negative social, economic, and health outcomes for both parents and children (Gipson et al., 2008). Contraception is an important tool in preventing unwanted pregnancies and achieving desired family size and birth spacing. In the U.S., approximately 65 % of women of reproductive age report currently using a method of contraception, and birth control pills are the most common method used by young adults (Daniels & Abma, 2020). While many studies have examined contraceptive use in the U.S., limited literature to date has explored contraceptive use among immigrants in general, and undocumented immigrants in particular.

Existing studies have found that immigrants have lower rates of accessing contraceptive healthcare and using contraception compared to non-immigrants, even after controlling for sociodemographic factors (Frost et al., 2021; Hasstedt et al., 2018; Shih et al., 2011; Tapales et al., 2018). In the only quantitative study to date of contraceptive use among Asian and Latinx undocumented immigrants, 88 % of respondents indicated they had unprotected sex in the past year (Sudhinaraset et al., 2022). This rate exceeds estimates among U.S. young adults generally, which suggest that about two-thirds report recently having unprotected sex (Jones et al., 2015; Nathan et al., 2023). Additionally, a majority of undocumented respondents in the study indicated that their immigration status impacted how they access sexual and reproductive healthcare, with barriers to care including cost of healthcare, not having health insurance, and not being comfortable seeking healthcare (Sudhinaraset et al., 2022). This indicates a need to examine access to contraceptive healthcare among undocumented immigrants.

The concept of access to healthcare is multi-faceted, incorporating awareness of the need for healthcare, acceptability of healthcare seeking, availability of healthcare providers, accessibility of healthcare facilities, and affordability of healthcare (Andersen, 1995; Levesque et al., 2013; Penchansky & Thomas, 1981). Thus, barriers to healthcare may include a lack of health knowledge, community norms discouraging healthcare use, limited providers of a particular specialty, geographic and transportation barriers to reaching healthcare facilities, and inability to pay for healthcare (Andersen, 1995; Levesque et al., 2013; Penchansky & Thomas, 1981). Undocumented immigrants may face many of these barriers to healthcare. In terms of contraceptive healthcare specifically, levels of health education regarding fertility and contraception, familial and cultural norms regarding use of sexual and reproductive healthcare, safety concerns regarding traveling to healthcare facilities and disclosing personal details to healthcare providers, and ability to pay for healthcare may condition healthcare access for undocumented immigrants.

Given that most contraceptive methods require a prescription from a healthcare provider, access to contraceptive healthcare is often required in order to use contraception. Studies have demonstrated that undocumented immigrants face several barriers to accessing healthcare, which may also apply to contraceptive healthcare. In particular, studies have emphasized that undocumented immigrants have limited access to health insurance and commonly face financial barriers to healthcare (Doshi et al., 2020; Hacker et al., 2015; Montealegre & Selwyn, 2014; Raymond-Flesch et al., 2014; Rodríguez et al., 2009; Sudhinaraset, Ling, et al., 2017a,b; Woofter & Sudhinaraset, 2022; Yu et al., 2020). While having insurance can improve access to healthcare, lack of knowledge about using insurance and difficulty navigating the healthcare system may pose barriers to accessing care even among insured undocumented immigrants (Doshi et al., 2020; Hacker et al., 2015; Montealegre & Selwyn, 2014; Raymond-Flesch et al., 2014; Rodríguez et al., 2009; Sudhinaraset, Ling, et al., 2017a,b; Woofter & Sudhinaraset, 2022; Yu et al., 2020). These trends have been demonstrated among both Asian and Latinx undocumented immigrants, though most studies focused on Latinx populations. While some studies suggest that having Deferred Action for Childhood Arrivals (DACA) status helps undocumented immigrants access care, others conclude that DACA alone is not sufficient to overcome these barriers (Raymond-Flesch et al., 2014; Sudhinaraset et al., 2017a,b; Woofter & Sudhinaraset, 2022). The lack of insurance coverage and lack of knowledge on navigating insurance may negatively impact access to contraceptive healthcare for undocumented immigrants, though studies are needed to understand the role of these and other barriers on contraceptive healthcare access.

Among undocumented immigrants who access healthcare, many report poor-quality care. For example, some Asian and Latinx undocumented immigrants report a lack of cultural competence among healthcare providers and even face discrimination when seeking healthcare (Doshi et al., 2020; Hacker et al., 2015; Raymond-Flesch et al., 2014; Rodríguez et al., 2009; Woofter & Sudhinaraset, 2022). Additionally, undocumented immigrants who do not speak English often experience language barriers and miscommunication with healthcare providers, particularly when translators are not available (Doshi et al., 2020; Hacker et al., 2015; Rodríguez et al., 2009; Woofter & Sudhinaraset, 2022). Language barriers are particularly common for Asian immigrants, as few medical staff and translators are available who speak Asian languages (Clough et al., 2013; Sudhinaraset et al., 2023). These experiences of poor-quality healthcare among undocumented immigrants may also apply to contraceptive healthcare, though no studies to date have explored these experiences.

### 1.1. Reproductive justice for undocumented women

The reproductive justice framework combines the principles of human rights, reproductive rights, and social justice (Ross et al., 2017). The three pillars of reproductive justice are the ability to have children, to not have children, and to raise children in safe environments (Ross et al., 2017). Achieving these three pillars for all people requires addressing barriers based on intersecting identities of race, gender, socioeconomic status, and immigration status, among others (Luna & Luker, 2013; Roberts, 1997; Ross et al., 2017). Notably, people of color and immigrants have experienced coercion to use contraception and not all individuals want to use contraception, nor should they be required to do so (Gomez & Wapman, 2017; Littlejohn, 2021; Oakley et al., 2018; Roberts, 1997; Rosenthal & Lobel, 2020). Still, access to high-quality reproductive healthcare that fully informs patients about their contraceptive options and provides autonomy in contraceptive decision-making is an important aspect of reproductive justice.

Threats to reproductive justice for immigrants date back to the earliest immigration policies in the U.S., and continue today with restrictions on health insurance coverage and other public benefits, lack of sexual and reproductive healthcare access in immigration detention, and family separation by immigration authorities, as well as anti-immigrant political rhetoric (Callaghan et al., 2019; Heffernan, 2023; Hiemstra, 2021; Huang, 2008; Messing et al., 2020; O’Leary & Simmons, 2017). The compounding effects of federal anti-immigrant policy, federal agency practice, and political sentiment result in complex barriers to achieving reproductive justice for undocumented immigrants in the U.S. Given the focus of anti-immigrant rhetoric and policy related to fertility specifically, undocumented immigrant women and others capable of pregnancy face particular pressure on their decision-making of when, whether, and in what context to have children (Abrego & Menjivar, 2011; Heffernan, 2023; Hiemstra, 2021; Huang, 2008; Sudhinaraset et al., 2025).

In addition to federal immigration policy in effect across the U.S., individual states also have immigrant policies which serve to include and/or exclude undocumented immigrants. For example, some states have inclusive policies providing access to public health insurance, driver’s licenses, and in-state tuition and financial aid for college to undocumented immigrants (De Trinidad Young & Wallace, 2019; Samari et al., 2021; Wallace et al., 2019). In contrast, some states also have exclusionary policies such as using E-Verify for employment hiring purposes and facilitating collaboration between local law enforcement and federal immigration enforcement (De Trinidad Young & Wallace, 2019; Samari et al., 2021; Wallace et al., 2019). The combination of these inclusive and exclusive policies are associated with health insurance status (Young et al., 2019), healthcare access (Dondero & Altman, 2020; Friedman & Venkataramani, 2021; Young et al., 2020), and health outcomes (Crookes et al., 2022; Sudhinaraset et al., 2021; Young et al., 2022) for immigrants. This state policy context further variegates undocumented immigrants’ ability to have children, not have children, and safely raise children depending on where they live within the U.S.

### 1.2. Objective

Despite the many barriers to healthcare and experiences of poor-quality healthcare among undocumented immigrants, limited literature has examined contraceptive healthcare in this population. The present study aims to help address this gap by exploring access to contraceptive healthcare and experiences with contraceptive healthcare among a sample of Asian and Latinx undocumented immigrant women in California. This study uses qualitative interviews to explore salient factors affecting access to and quality of contraceptive healthcare in this population. This study also highlights barriers to contraceptive healthcare and experiences of poor-quality contraceptive healthcare, which can help inform future efforts to ensure access to desired contraception and high-quality contraceptive care for undocumented immigrants.

## 2. Methods

### 2.1. Study setting and population

This study is based in California, which is both home to the largest population of undocumented immigrants of any state (Passel & Krogstad, 2024) and is one of the most inclusive states based on state immigrant policy context (Samari et al., 2021; Wallace et al., 2019). Over the past decade, California has continually expanded access to state Medicaid coverage – known as MediCal – for undocumented immigrants (Cha & McConville, 2021). As of 2024, California allows undocumented immigrants of all ages to access MediCal (McConville & Mustala, 2024). Additionally, California funds free contraceptive services at select healthcare facilities for low-income Californians regardless of documentation status through the Family Planning, Access, Care, and Treatment (Family PACT) program (Cha & McConville, 2021).

This study focuses on both Asian and Latinx undocumented immigrants, which represent the two largest racial/ethnic groups of undocumented immigrants (Passel & Krogstad, 2024; Profile of the Unauthorized Population: United States, 2024). While the majority of studies of undocumented immigrants have focused exclusively on those descending from Latin America, scholars have called for additional research on undocumented Asian immigrants (Nayak et al., 2024). For example, studies have demonstrated that Asian undocumented immigrants face particular stressors based on stigma and low social capital (Sudhinaraset et al., 2017a,b).

### 2.2. Data collection

This project leverages 24 in-depth interviews conducted as part of the multi-wave BRAVE Study (Building communities, Raising All immigrant Voices for health Equity) between April and July 2022 with Asian and Latinx undocumented immigrant women in California. Details of this study have been published previously (Leon et al., 2025; Sudhinaraset et al., 2025). Though six additional participants completed interviews, they indicated they had not ever had sexual intercourse, and thus were excluded from the current analysis. Interview participants were recruited through outreach to prior respondents to earlier BRAVE surveys who were willing to be re-contacted as well as through advertisements with local organizations serving undocumented immigrants, social media platforms, and the personal networks of the study team. Eligible participants included those who identified as women, were currently or formerly undocumented, identified as Asian and/or Latinx, were of reproductive age (between 18 and 40 years old), and lived in California at the time of the interview. Interviews were offered in both English and Spanish. Participants gave verbal informed consent prior to beginning the interview. Participants received $40 gift cards following the interviews in appreciation of their time. This study was approved by the Institutional Review Board at the University of California, Los Angeles (IRB #20–000972).

Interviews were conducted via Zoom by four female Asian and Latinx researchers who had personal connections to immigration and were experienced in qualitative interviewing. Each interviewer completed a two-day training on qualitative research, immigration, and sexual and reproductive health. Interviewers also completed practice pilot interviews prior to beginning data collection. The study team collaboratively created a field guide to use during interviews which was revised and updated throughout data collection as needed. The field guide included questions about participants’ experiences immigrating to the U.S., any changes in their immigration status over time (including receiving DACA), childhood experiences, family relationships, education, employment, romantic relationships, sexual activity, fertility intentions, and experiences with sexual and reproductive healthcare including contraceptive use (see Appendix A). A demographic form also collected information such as participants’ age, country of origin, and education level. Interviews lasted approximately 1 hour and all interviews were recorded and transcribed for analyses. Any interviews conducted in Spanish were first transcribed and then translated to English by the interviewer. All transcripts were reviewed for accuracy and de-identification prior to analyses.

### 2.3. Data analysis

After all interviews were conducted, a team of three researchers, including two of the interviewers, inductively coded the transcripts. An iterative approach was used to create and revise a codebook that accurately and comprehensively captured the sentiments expressed by participants. Definitions for each code and determinations of how to apply each code were made by consensus amongst the coders. Coding took place over four phases, with the coders meeting after each phase to discuss any questions and to update the codebook as needed. In the first phase, two coders independently coded the same five transcripts, developing a list of relevant codes as they coded. A third coder then reconciled the two versions of these five transcripts to create an initial codebook. In the second phase, the three coders each coded five different transcripts and reviewed each other’s coded transcripts, noting any questions and suggestions. The codebook was then revised based on all new codes identified during the second phase. In the third phase, one coder coded the remaining 10 transcripts and finalized the codebook. Finally, in the fourth phase, one coder reviewed all 30 transcripts to ensure that all codes in the final codebook were consistently applied to all transcripts. The list of parent codes from the final codebook are included in Appendix B. The integration of several coders and an emphasis on consistency in coding was prioritized by the study team to ensure that the coding scheme captured all relevant sentiment expressed by participants across the many topics covered in interviews, and allowed the large study team to leverage the coded interviews for multiple different analyses. All coding was conducted using Dedoose qualitative software.

After coding was complete, excerpts under codes related to contraceptive use and contraceptive healthcare were reviewed by the authors to address the study aims. The authors used thematic analysis to identify themes related to access and barriers to contraceptive healthcare and experiences with contraceptive healthcare including the quality of healthcare received (Braun & Clarke, 2006, 2014). Themes were also examined for any differences or patterns between Asian and Latinx participants, though all identified themes were expressed by both Asian and Latinx participants with no clear differences.

## 3. Results

### 3.1. Sample overview

Table 1 depicts the characteristics of the sample. Of the 24 participants in the analytic sample, 15 identified as Latinx and 9 identified as Asian. The majority of Latinx participants were from Mexico and two were from Central America. The majority of Asian participants were from South Korea and two were from Southeast Asia. One interview was conducted in Spanish and the rest were conducted in English. Participants ranged in age from 20 to 37, and most were between 25 and 29 years old. Three-quarters of participants held a four-year college degree or above and three-quarters of participants were DACA recipients. At the time of the interviews, two-thirds of participants were in a romantic relationship (including those who were married) while one-third were not in a romantic relationship. One-quarter of participants had one or more children. No interviewees were pregnant at the time of the interviews and none were currently intending to become pregnant.

All 24 participants had used some form of contraception including condoms at least once in their lives, primarily based on a desire to avoid pregnancy. Nearly two-thirds of participants had used oral contraceptives and over one-third had used intrauterine devices (IUD) or implants. Three participants had used contraceptive injections and/or patches. Additionally, one-quarter of participants had used emergency contraception such as Plan B. One participant noted that the only contraceptive method she had ever used was condoms, and two other participants had only ever used condoms and emergency contraception.

Table 2 lists the six themes identified from the interviews with example quotes for each theme. Factors impacting access to contraceptive healthcare included (1) knowledge about contraception, (2) health insurance coverage, and (3) health insurance knowledge. The quality of contraceptive healthcare participants experiences was described as (4) judgmental or dismissive, (5) cursory or impersonal, or (6) person-centered. Each theme is described below with illustrative quotes.

### 3.2. Access to contraceptive healthcare

Participants described three main factors impacting access to contraceptive healthcare: knowledge about contraception, health insurance coverage, and knowledge about using health insurance. Some participants reported having little knowledge about contraception, largely due to a lack of comprehensive sex education, while others were concerned for side effects or complications of using contraception. Still, some participants learned about accessing contraceptive healthcare from friends or family which helped them access care. Nearly all participants who accessed contraceptive healthcare had health insurance at the time, often due to their status as college students, though many faced periods of uninsurance. However, some participants who had insurance lacked knowledge of how to use their health insurance to access contraceptive healthcare.

#### 3.2.1. Knowledge about contraception

Many participants noted that they lacked comprehensive, accurate knowledge regarding contraception. Participants often indicated that they never learned about contraception methods due to lack of sex education while growing up. One participant shared:

“So the first time I became sexually active was in high school. And I think at that point, I didn’t really have an idea of any sort of, like, what all the different types of contraception are. I actually just knew, like, the condom, to be honest. And that was what we used.” (27-year-old Latinx)

Some participants chose not to seek contraceptive healthcare because their concerns about potential side effects outweighed their desire to use contraception. One participant shared:

“I’ve never been on birth control because I hear that you gain weight … it’s kind of dumb looking back at it now, what do you want, weight or a baby? But um, so I’ve never felt comfortable going to get it.” (30 year-old Latinx)

Other participants were concerned about possible adverse outcomes of contraception which made them choose not to seek contraceptive healthcare. For example, some participants were unsure about the safety of different contraceptive methods and were afraid that using contraception could threaten their ability to have children in the future. One participant described a fear of uterine cysts as a result of contraceptive use based on information from friends:

“I’m not comfortable [using contraception] because I don’t know what it’s going to do to my body. And I would just rather not go that route. I’ve had friends who have gone through the birth control route and have had severe cysts on their uterus and have had to go through surgery for that. I know everybody’s body is different, but I just think I’m just scared to do something like that.” (27 year-old Asian)

Other participants believed that they were unlikely or unable to become pregnant, and thus did not need contraception. One participant described an unintended pregnancy that resulted from unprotected sex when she believed she was unable to get pregnant:

“When I had my first baby, she was not planned. It was not planned. I just felt that because of my reproductive system, like, me having an abnormal period and my previous relationship that I never had taken care of myself or used any contraceptives, I’m like, okay, well, you know what, I’m just not fertile. I’m not gonna get pregnant.” (37-year-old Latinx)

However, some participants noted that they obtained useful information about seeking contraceptive healthcare from their older friends or family members. One participant described learning about a local clinic that provides contraceptive healthcare without requiring parental involvement, which helped her to access contraception as a teenager:

“There’s this clinic that’s close to my house. It’s like, a medical clinic. And my cousin actually told me about it in high school, that she went there … you know, we were younger, [and] they don’t ask for your parents or anything … So I went there because of her.” (28 year-old Latinx)

#### 3.2.2. Health insurance coverage

Nearly all participants who used prescription-based methods of contraception had insurance at the time they accessed contraceptive healthcare, including having public insurance (MediCal) or private insurance through college, employment, or their parents. This health insurance was pivotal for accessing contraceptive care, and many participants used contraception for the first time once they became eligible for health insurance coverage. One participant shared:

“As soon as I knew I had health insurance, the first thing I wanted to do was go get birth control.” (26 year-old Latinx)

Notably, college was the first time that many participants had health insurance coverage and convenient access to healthcare, and thus was the first time they accessed contraceptive healthcare. One participant attributed getting contraception for the first time to having access to student health services where contraecptive healthcare was covered by student health insurance:

“I don’t think I would’ve done that [gotten an IUD] if I didn’t have the ease of a student health center.” (28-year-old Latinx)

However, several participants faced barriers to contraceptive care related to health insurance. When participants were uninsured, generally either prior to college or after graduating college, they described a lack of access to contraceptive healthcare. One participant described initially beginning birth control pills as a teenager, but stopping shortly after because she did not have insurance and her family could not afford to continue paying for the prescription:

“I’ve been, like, on birth control since I was like 15 … so I started with that [birth control pills], but I was really bad at taking it. So I kind of stopped taking them. And, you know, I feel like we just never went back to the doctor around that time because it was expensive. We didn’t have insurance. So we just let it go.” (27-year-old Latinx)

Similarly, some participants who were not insured relied on state programs that provide free access to contraception. One participant described being told at a healthcare clinic that she was not eligible for the program, and thus she did not receive the contraceptive healthcare she desired at that time:

“The first time I went to [reproductive health clinic], they said I was ineligible for, like, the free services. And I was like, there’s no way I can pay for this. I didn’t have health insurance at the time … But they said I don’t qualify for free coverage, so they turned me away.” (29-year-old Asian)

Furthermore, some participants made contraceptive decisions anticipating being uninsured in the future, such as selecting a method that would last for several years without requiring ongoing contraceptive healthcare. One participant described her contraceptive decision-making as she was aging out of the health insurance coverage she had through her parents:

“I’m currently insured because of my dad’s job. I know that that won’t any longer be the case when I turn 26. So last summer I decided to get an IUD, because I just wanted to have coverage I guess for, like, a while, since it’s something that can last for, like, six years.” (24-year-old Latinx)

One participant experienced uninsurance after becoming unemployed and was unable to access contraceptive healthcare. She noted that she was only able to keep using her contraceptive method during this period because she had inconsistently used birth control pills in the past and had saved the unused pills:

“There were a few months that I had no [health insurance] coverage. I accessed [birth control] pills by just using the ones I had stockpiled. There were some months that, I don’t remember why, but I think I was not seeing anyone and not having sex … so I think I had maybe three months’ worth of pills … so when I became unemployed, I used that.” (24-year-old Asian)

#### 3.2.3. Knowledge about using health insurance

Some participants did have insurance which they could have used to access contraceptive healthcare, but did not know how to navigate the insurance process, and thus did not access contraceptive healthcare. Although these participants had health insurance, it did not help them access healthcare as they were unable to navigate the health insurance process. One participant described not knowing how to use insurance until she went to graduate school:

“Before graduate school, I mean, I had insurance, I just never really figured out how to use it. I think growing up undocumented, no one told me this is how I use insurance.” (28-year-old Latinx)

Similarly, one participant described having insurance while in college, but never learning how to find a healthcare provider who accepted that insurance:

“I remember I paid a lot of money for health insurance through the school [college], and I never used it because I was kind of intimidated by, like, the PPO [preferred provider organization] system, where you, like, contact people and ask if they’re taking new patients.” (28-year-old Asian)

### 3.3. Quality of contraceptive healthcare

Participants who were able to access contraceptive healthcare had varying experiences with healthcare providers when seeking contraception. The contraceptive healthcare experiences described are categorized below into judgmental or dismissive, cursory or impersonal, and person-centered based on participants’ descriptions of the care they received.

#### 3.3.1. Judgmental or dismissive contraceptive healthcare

Many participants described experiences with contraceptive healthcare in which providers were judgmental and dismissed their concerns regarding contraceptive methods. For many participants who reported this type of care, these experiences resulted in them not receiving any contraception at the time of the healthcare visit as well as not wanting seek contraceptive care in the future. One participant described feeling judged by a healthcare provider when she first sought contraception due to the fact that she had not ever used contraception before:

“The first time I went to the health center through my school [college], I felt a little judged by the doctor when I started asking about birth control pills. I remember she asked me, like, ’well, are you on birth control now?’ And I mentioned, like, no, and she said ‘well, do you want to get pregnant or what?’ And I was just like ‘oh my god, well no, that’s why I’m here!’ So yeah, so it was just, like, very rude … I felt judged.” (27-year-old Latinx)

Another participant shared that she had previously become pregnant while using an IUD, and had concerns about relying on this method again, which were dismissed by her provider:

“I also don’t know if I’m just, like, extra sensitive because of it [previously getting pregnant while using an IUD], but I just kind of feel like sometimes my gynecologist, like, dismisses my concerns. Because I feel like I have these concerns for a reason, like, I trusted that my IUD would do the job and it didn’t … but she [gynecologist] is always like, you know, ‘IUDs do this, IUDs do that, like it’s normal.’ And like, I feel like she’s – I get to some [extent] she’s trying to, like, reassure me, but it sometimes comes off kind of like she’s dismissing a lot of the concerns that I have.” (38-year-old Asian)

#### 3.3.2. Cursory or impersonal contraceptive healthcare

Some participants described experiences with contraceptive healthcare that were cursory or impersonal. These participants generally received the contraceptive method they were seeking, but did not feel their providers were particularly helpful or warm, and often did not receive comprehensive information about the contraceptive method they chose. Although these participants received contraception, these healthcare experiences were missed opportunities to address any questions and concerns participants had and to foster trust in healthcare for the future. One participant noted that her provider did not take the time to build a relationship with her and provided only cursory information regarding how to use the contraceptive method she had chosen:

“I didn’t really have a connection or a relationship with the doctor. She was just like, ‘okay, well, this is how you’re going to take it [birth control pills]. This is what you’re going to do. This is what it does. These are the symptoms. These are the side effects.’ And there you go, off you go, kind of.” (22-year-old Latinx)

Another participant described her experience with impersonal care when she sought contraception:

“I guess they treated me fairly. I guess she just, like, asked me what I was there for. Like, it was just, like, very to the point … she didn’t really go into much detail about anything. It was just like oh, here it is, and then goodbye. Like, it wasn’t very personal.” (28-year-old Latinx)

#### 3.3.3. Person-centered contraceptive healthcare

Finally, some participants described person-centered contraceptive healthcare experiences in which providers fully explained contraceptive options without judgement and provided autonomy in contraceptive decision-making. These positive experiences led to participants making fully-informed decisions about contraception and using their desired method of contraception at the time of the healthcare visit. These experiences also made participants feel comfortable continuing to seek healthcare in the future. One participant shared:

“I got contraception through college. I was on the pill and they had, not an OBGYN [obstetrician/gynecologist] but an RN [registered nurse], that was very knowledgeable about talking to college kids who were using contraception for the first time. She was the first person that actually explained how it [contraception] works to me, because I don’t think they said in high school, really. I don’t remember learning anything … I had no idea how the [birth control] pill worked. And she explained that, and that was really, really helpful.” (24-year-old Asian)

Similarly, another participant appreciated that her healthcare provider fully educated her about contraception and gave her time to make the best decision for her:

“I went into that doctor’s office and I was like, ’I know nothing about contraception. Like, I know nothing about birth control’ … She pulled out a booklet and we looked at all of them [contraceptive methods] and we looked at my options. And you know, she explained things to me, she gave me things to read, and didn’t, like, force me to make a decision on the spot.” (26-year-old Latinx)

Indeed, another participant noted that her provider took her seriously, treated her with respect, and gave her all of the necessary information to make an informed decision:

“Never did she [healthcare provider] make me feel uncomfortable or bad or anything. She never judged me. She just wanted to help me and for me to be protected. When I came in, she didn’t treat me like a little girl, you know, like, ‘where are your parents?’ or something … She gave me options. The first time I went I remember she told me, you know, ‘I’m not sure what you are looking for, but we have this and this’ … she gave me knowledge and information on everything.” (28-year-old Latinx)

## 4. Discussion

The current study builds upon existing literature on the multifaceted barriers to healthcare and experiences with healthcare among undocumented immigrants by examining access to and quality of contraceptive healthcare among Asian and Latinx undocumented immigrants in California. Despite all participants in this study living in an inclusive immigrant state and the majority of participants having college degrees and DACA status, most participants struggled to access contraceptive healthcare at some point and many reported negative experiences with contraceptive healthcare. These findings reinforce the need to consider the unique threats to reproductive justice for undocumented immigrant women.

Undocumented immigrant participants in this study reported barriers to contraceptive healthcare including lack of knowledge regarding contraception, lack of health insurance coverage, and lack of knowledge about using health insurance. In this study, several participants indicated that they had not received comprehensive sex education and had little knowledge of contraceptive methods. While some had concerns about side effects than can occur with contraception, others held misconceptions about adverse consequences of contraceptive use which made them avoid contraception. Several studies have noted that Hispanic immigrants report low levels of contraceptive education and knowledge (Batek et al., 2024; Craig et al., 2014; Gozzi et al., 2024; Quelopana & Alcalde, 2014; Villar & Concha, 2012; White et al., 2017), including high levels of contraceptive misinformation (Garcés-Palacio et al., 2008; Gozzi et al., 2024) and low perceived likelihood of becoming pregnant (Gemmill et al., 2021). One study among South Asian immigrants also highlighted a need for more contraceptive education (Farid et al., 2013). Education regarding fertility and contraception may be useful for undocumented immigrants to help make fully-informed decisions about whether to use contraception and which method may best fit their needs and preferences. Still, even with full knowledge, some side effects and risks are inherent with contraceptive use, and not all undocumented immigrants will be comfortable using contraception, which should also be respected.

Health insurance is another key factor impacting access to contraceptive healthcare among undocumented immigrants. Existing studies have also demonstrated the importance of health insurance for healthcare access among immigrants across documentation statuses (Doshi et al., 2020; Gozzi et al., 2024; Hacker et al., 2015; Montealegre & Selwyn, 2014; Raymond-Flesch et al., 2014; Rodríguez et al., 2009; Sudhinaraset et al., 2022). Notably, undocumented immigrants are unable to access public or private health insurance in the majority of states, and an estimated 50 % of undocumented immigrants do not have health insurance (Key Facts on Health Coverage of Immigrants, 2025; Pillai et al., 2024). Although most participants in this study had been insured at some point, most participants had also experienced periods of uninsurance. In this way, policies providing insurance coverage for undocumented immigrants may not prevent these individuals from experiencing periods of uninsurance. Additionally, college was a pivotal time for many participants to have both health insurance and convenient access to healthcare providers through student health programs, and many participants accessed contraception for the first (and sometimes only) time during this period. California is one of few states that provide in-state tuition and financial aid to undocumented college students, which may help facilitate access to contraceptive healthcare in this population (Samari et al., 2021). Finally, even when policies provide health insurance for undocumented immigrants, additional efforts are needed to ensure that they also have sufficient information on how to use health insurance to access healthcare.

Notably, although significant literature has found that fear of being stopped by police when traveling to healthcare facilities or fear that personal information disclosed at healthcare facilities may then be shared with law enforcement or immigration authorities act as barriers to healthcare among undocumented immigrants (Callaghan et al., 2019; Clough et al., 2013; Doshi et al., 2020; Hacker et al., 2015; Montealegre & Selwyn, 2014; Raymond-Flesch et al., 2014; Sudhinaraset, Ling, et al., 2017; Yu et al., 2020), this theme did not emerge in the current study. This may be due to the inclusive policy environment for immigrants in California (Samari et al., 2021; Wallace et al., 2019) and the high rates of DACA status in this sample, which confers some protection from deportation. Future research should examine this in samples across state immigrant policy contexts and DACA statuses.

Participants in this study described a range of experiences during contraceptive healthcare, from judgmental to cursory to person-centered. In the U.S., people of color and immigrants report experiencing poor-quality contraceptive healthcare and discrimination when seeking contraception (Boniface et al., 2024; Gomez & Wapman, 2017; Gozzi et al., 2024; Oakley et al., 2018; Rosenthal & Lobel, 2020; Welti et al., 2022; Wingo et al., 2023). Further, negative experiences and discrimination by healthcare providers are common among undocumented immigrants and lead some to avoid healthcare in the future (Doshi et al., 2020; Hacker et al., 2015; Raymond-Flesch et al., 2014; Sudhinaraset, Ling, et al., 2017; Woofter & Sudhinaraset, 2022). The current study expands these findings, noting that some undocumented immigrants experience poor-quality contraceptive healthcare specifically. The negative experiences with contraceptive healthcare described in this study were not only associated with not receiving contraception at the healthcare encounter, but also fostered hesitation to seek contraceptive healthcare in the future. In this way, negative experiences with contraceptive healthcare became an additional barrier to accessing care. Even experiences of contraceptive healthcare that were cursory or impersonal, as experienced by several participants in the current study, represent missed opportunities to increase knowledge regarding contraception and to build relationships with patients which can facilitate continuity of reproductive healthcare. Though it not clear from this study the extent to which negative contraceptive healthcare experiences were driven by participants’ documentation status versus their race/ethnicity, this should be explored further in future research.

Notably, though, some participants in this study reported experiencing person-centered contraceptive healthcare in which providers gave full information without judgment and allowed patients autonomy in their contraceptive decision-making (Sudhinaraset, Afulani, et al., 2017). These participants both received their contraceptive method of choice during the healthcare encounter and were empowered and motivated to seek contraceptive healthcare in the future. High-quality person-centered contraceptive healthcare is vital to facilitate access to contraception for undocumented immigrants, including providing education on contraceptive methods and proper use of contraceptives, as well as building trust in healthcare more broadly. Future work could leverage quantitative data and the Person-Centered Contraceptive Counseling measure to examine levels of person-centered care for undocumented immigrants (Boniface et al., 2024; Dehlendorf et al., 2023; Wingo et al., 2023).

### 4.1. Limitations, strengths, and future directions

The primarily limitation of this study is the unique sample of undocumented immigrants. Participants in this study all lived in California, an inclusive state for undocumented immigrants. Although participants were only required to live in California at the time of the interview, most participants had lived for several years or even their entire lives since immigrating in California. Few participants had ever lived in other U.S. states. Additionally, most participants had college degrees and DACA status. As a result, undocumented immigrants living in more restrictive contexts and those with less education and without DACA status may face additional barriers to contraceptive healthcare not discussed here, particularly related to fear of immigration enforcement, as well as more negative contraceptive healthcare quality. By focusing on this sample, this study highlights the level of access to contraceptive healthcare and experiences with contraceptive healthcare among those undocumented immigrants with relatively high levels of healthcare access. Notably, prior studies have found that while DACA status confers some improvement in socioeconomic status, access to healthcare, and health outcomes (Hamilton et al., 2020; Patler et al., 2020; Sudhinaraset et al., 2017a,b; Woofter & Sudhinaraset, 2022), the uncertainty regarding the future of the DACA program causes stress and limits its usefulness for recipients (Aranda et al., 2023; Patler et al., 2019; Zarate et al., 2024). Similarly, this study shows that DACA may help undocumented immigrants access contraceptive healthcare, but does not entirely overcome barriers to care.

Despite these limitations, this study is strengthened by its inclusion of both Latinx and Asian undocumented immigrants. Asian immigrants are not often included in studies of the health of undocumented immigrants, despite being the second largest group of undocumented immigrants in the U.S. (Passel & Krogstad, 2024). Existing research suggests that while Latinx immigrants may experience higher levels of policy exclusions compared to Asian immigrants, these groups have similar healthcare access and health outcomes based on historical treatment and policies against Asian immigrants (Young et al., 2023). This study responds to the calls of scholars for more data examining the health of Asian undocumented immigrants (Nayak et al., 2024). This is also one of the first studies to examine contraceptive healthcare access and quality among undocumented immigrants. Given that approximately two-thirds of undocumented immigrants are of reproductive age and nearly half are female, this is an important area of research (Profile of the Unauthorized Population: United States, 2024).

Future research should build upon the findings described here to further investigate access to and quality of contraceptive healthcare among undocumented immigrants. Additional studies should leverage both qualitative and quantitative research methods to assess the frequency of different barriers to contraceptive healthcare across the undocumented population. Studies should also investigate differences across subgroups based on race/ethnicity, socioeconomic status, and DACA status in diverse samples. Studies that examine experiences across states with differing state immigrant policy climates would also provide useful nuance. Future research should also examine the extent to which contraceptive healthcare for undocumented immigrants is person-centered and salient aspects of contraceptive healthcare in this population. Additionally, research that identifies policies and interventions to increase contraceptive education, consistent health insurance coverage, knowledge regarding health insurance, and quality of contraceptive healthcare provided to undocumented immigrants can help work towards reproductive justice for undocumented immigrants.

### 4.2. Conclusion

This study is one of the first to investigate contraceptive healthcare access and quality among undocumented immigrants. This study identified several relevant barriers to contraceptive healthcare, including lack of contraceptive knowledge, lack of insurance coverage, and lack of knowledge about using health insurance. Additionally, this study noted that while some participants experienced person-centered contraceptive healthcare, others reported contraceptive healthcare that was impersonal or cursory and even judgmental or dismissive. Given that most participants in this study had college degrees, DACA status, and lived in an inclusive state policy climate, the barriers to contraceptive healthcare and negative care experiences faced by less privileged undocumented immigrants undoubtedly exceed those noted here. These findings demonstrate the need for policies and programs to improve contraceptive education and expand insurance coverage and knowledge for undocumented immigrants. Additionally, healthcare providers should provide high-quality, person-centered contraceptive healthcare to undocumented immigrants, including taking the time to educate patients on contraception and support patients in making fully informed decisions regarding contraceptive use. These additional efforts are necessary to work towards reproductive justice for undocumented immigrant women in the U.S.

## Supplementary Material

Supplementary Material

## Figures and Tables

**Table 1:** Characteristics of in-depth interview participants in the analytic sample (N = 24).

Variable	N (%)

**Race/Ethnicity**	
*Asian*	9 (38 %)
*Latinx*	15 (63 %)
**Age**	
*20–24*	5 (21 %)
*25–29*	14 (58 %)
*30+*	5 (21 %)
**Education**	
*High school or less than high school*	5 (21 %)
*Completed college or above*	19 (79 %)
**Relationship Status**	
*Not in an relationship*	8 (33 %)
*In a relationship or married*	16 (67 %)
**Parity**	
*No children*	18 (75 %)
*One or more children*	6 (25 %)
**Deferred Action for Childhood Arrivals (DACA) Status**	
*Not DACA recipient*	6 (25 %)
*DACA recipient*	18 (75 %)

**Table 2:** Identified themes from in-depth interviews with example quotes.

Theme	Example Quote

**Access to Contraception**	
*Knowledge about contraception*	“When I had my first baby, she was not planned. It was not planned. I just felt that, I just felt that because of my reproductive system, like, me having an abnormal period and my previous relationship that I never had taken care of myself or used any contraceptives, I’m like, okay, well, you know what, I’m just not fertile. I’m not gonna get pregnant.” (37-year-old Latinx)
*Health insurance coverage and knowledge*	“The first time I went to [reproductive health clinic], they said I was ineligible for, like, the free services. And I was like, there’s no way I can pay for this. I didn’t even know what that meant. So I didn’t have health insurance at the time ... But they said I don’t qualify for free coverage, so they turned me away.” (29-year- old Asian)
*Knowledge about using health insurance*	“Before graduate school, I mean, I had insurance, I just never really figured out how to use it. I think growing up undocumented, no one told me this is how I use insurance.” (28-year-old Latinx)
**Quality of Contraceptive Healthcare**	
*Judgmental or dismissive*	“The first time I went to the health center through my school [college], I felt a little judged by the doctor when I started asking about birth control pills. I remember she asked me, like, ’well, are you on birth control now?’ And I mentioned, like, no, and she said ‘well, you want to get pregnant or what?’ And I was just like ‘oh my god, well no, that’s why I’m here!’ So yeah, so it was just, like, very rude ... I felt judged.” (27-year-old Latinx)
*Cursory or impersonal*	“I guess they treated me fairly. I guess she just, like, asked me what I was there for. Like, it was just, like, very to the point ... she didn’t really go into much detail about anything. It was just like oh, here it is, and then goodbye. Like, it wasn’t very personal.” (28-year- old Latinx)
*Person-centered*	“Never did she [healthcare provider] make me feel uncomfortable or bad or anything. She never judged me. She just wanted to help me and for me to be protected. When I came in, she didn’t treat me like a little girl, you know, like, ‘where are your parents?’ or something ... She gave me options. The first time I went I remember she told me, you know, ‘I’m not sure what you are looking for, but we have this and this’ ... she gave me knowledge and information on everything.” (28-year-old Latinx)

## Appendix A and Appendix B Supplementary data. Supplementary data

Supplementary data to this article can be found online at https://doi.org/10.1016/j.ssmqr.2025.100655.
