# Supplementary material for: Access to and quality of contraceptive healthcare for undocumented immigrant women in California
Source: SSM Qual Res Health. Author manuscript; Available in PMC 2026 Jun 27. (PMC13308633; doi:10.1016/j.ssmqr.2025.100655)
Supplement: Supplementary Material [file NIHMS2174094-supplement-Supplementary_Material.docx]

**Appendix A. Life History Interview Field Guides**

**Interview Part 1 — Immigration History**

I wanted to start by learning more about you and your immigration story.

1. Can you tell me about your life before moving to the US?
2. What do you remember about your first move to the US?
3. What was it like growing up in the US?
4. Could you walk me through the changes in immigration status(es) you’ve had since entering the US?
5. Have you had any opportunities to change immigration status(es) since moving to the US?
6. How do you feel about your right to live in the US? What has influenced these feelings?

**Interview Part 2 — SRH Services**

Next I want to take some time to talk about your experiences in accessing sexual and reproductive health services.

Please mark all that apply with an (X).
You can mark more than one choice (A, B, C, and/or D) for each row (#1-12).

| Service | A. I have accessed this at least once in my life | B. I have tried to access this but could not access it at least once in my life | C. I did NOT try to access this at least once in my life | D. Don’t know or not applicable |
| --- | --- | --- | --- | --- |
| 1. Contraception |  |  |  |  |
| 2. Pap smear |  |  |  |  |
| 3. Pelvic exam |  |  |  |  |
| 4. STI testing |  |  |  |  |
| 5. STI counseling |  |  |  |  |
| 6. Pregnancy test |  |  |  |  |
| 7. Prenatal care |  |  |  |  |
| 8. Postpartum care |  |  |  |  |
| 9. Abortion services |  |  |  |  |
| 10. HPV vaccine #1 |  |  |  |  |
| 11. HPV vaccine #2 |  |  |  |  |
| 12. HPV vaccine #3 |  |  |  |  |

1. I saw in the chart that you indicated… Can you tell me more about…
   1. If participant marked (A):
      1. What was your experience like?
      2. Where did you go?
      3. Did the provider treat you with respect? What does that look like?
      4. How much did you trust your provider? Why?
      5. How did you pay for your visit?
   2. If participant marked (B):
      1. Can you tell me what prevented you from accessing the service?
   3. If participant marked (C):
      1. Can you tell me why you chose not to access the service?
2. What contraceptive methods have you used?
   1. How did you obtain them?
   2. When did you use them?
   3. How do you decide?
   4. Do you feel that they are effective?
   5. If you have not used any, why did you make that decision?
3. [If participant has ever accessed abortion]
   1. Did you face any barriers to abortion care?
   2. What are your family’s attitudes regarding abortion? How about other people?
   3. Did your immigration status affect your decision making or care seeking process at all?
   4. How did you feel before, during, and after your abortion?
4. Thinking about times when you had or didn’t have insurance, how did your sexual and reproductive healthcare access change?
5. Do you want to talk about any other sexual and reproductive health or healthcare experiences that I may not have covered?
6. How has the pandemic impacted your access to sexual or reproductive health services, if at all?
7. How does your immigration status influence your perceptions around abortion services?
   1. [Added in April 2022] How might the changing laws around abortion impact undocumented immigrants in particular?

**Interview Part 3 — Relationships**

Now I would like to ask you about your romantic partners, relationships, and experiences.

1. Have you ever had a romantic relationship?
   1. Did you disclose your immigration status to your partner? How did that go?
   2. What are some ways your partner made you feel safe or supported as an undocumented person? Were there ways they ever made your feel unsafe or unsupported?
   3. What are your thoughts around marriage?
2. Does immigration status affect how you select or engage with romantic partners?
   1. Have you ever felt pressured to stay in or do something in a relationship because of your immigration status?
3. Was dating ever encouraged, discouraged, or influenced by anyone or anything?
4. Have you ever thought about having children?
   1. How does immigration status influence your decision to have or not to have any children?
   2. Have you experienced pressure to have or not have any children?
   3. Does your status influence any hopes that you have for your future family?

Next, we’re going to move onto questions about sexual experiences. I understand if some of the questions might feel uncomfortable to think about or discuss. If, at any point, you do not want to answer, want to take a break, or stop the interview, please let me know and I can pause or stop recording.

1. Have you ever had sexual intercourse?
   1. At what age(s)?
   2. What were your pregnancy intentions at the time?
   3. Were you able to use any contraception or practice any birth control at the time?
2. Who do you talk to about sex, sexual and reproductive health, or other related matters?
   1. Probe: Partners, family, friends, professionals?
   2. What makes them trustworthy?

Next I am going to ask you questions about sexual coercion, which may also elicit some discomfort. I just want to remind you again that you are also in control of our conversation and if at any point, you want to discuss something else, do not want to answer, pause, or stop the interview, you can feel free to interrupt and let me know.

1. Have you ever experienced any unwanted sexual activity?
   1. At what age(s)?
   2. To the extent that you feel comfortable, can you tell me what happened?
   3. How did you want to respond to the incident?
      1. What allowed you to do so? What prevented you from doing so?
      2. [If law enforcement is mentioned]: What is your level of trust in law enforcement?
   4. Has anybody counseled you about applying for a U or T visa through the Violence Against Women Act (VAWA)?

**Interview Part 4 — Resilience**

Next I want to talk about resilience and the ways in which your community has adapted to challenges.

1. How do you define sexual and reproductive health?
2. What does reproductive justice mean to you?
3. When you picture your ideal future family, what would that look like?
4. What sources of strength, resources, or services do people leverage or rely on to meet their reproductive health needs?
   1. What tactics or strategies are supportive in this?
5. Do you have trusted sources of healthcare in your community?
   1. If yes, what makes them trustworthy?
6. What recommendations do you have to improve access to and quality of sexual and reproductive healthcare services for other undocumented immigrants?

**Appendix B. Parent codes from final codebook**

- Immigration Decision-Making and Experience
- Immigration Statuses and Changes
- Life in United States
- Education
- Employment
- Insurance Status
- Experience with Institutions and Resources
- Experiences with Police/Immigration Enforcement
- Friends and Community
- Family Relationships and Dynamics
- Romantic Relationships
- Sexual Activity
- Sexual Violence
- Pregnancy/Fertility Intentions
- Health and Healthcare (Not Sexual & Reproductive)
- Mental Health and Wellbeing
- Views on Sexual and Reproductive Health
- Contraception and Abortion Healthcare and Experiences
- Pregnancy Healthcare and Experiences
- Sexually Transmitted Infection Healthcare and Experiences
- Quality of Sexual and Reproductive Healthcare
- Resilience
- Recommendations
